# Idiopathic pneumonia syndrome after bone marrow transplantation presenting with "crazy-paving" pattern on high-resolution computed tomography: a case report

**DOI:** 10.1186/1757-1626-1-234

**Published:** 2008-10-13

**Authors:** Taisa Davaus Gasparetto, Edson Marchiori, Marina B Guimarães, Dante Luiz Escuissato, Gláucia Zanetti

**Affiliations:** 1 Department of Radiology of the Fluminense Federal University, Rua Marquês do Paraná, 530. Centro, CEP 24.000.000, Niterói, Rio de Janeiro, Brazil; 2 Department of Radiology of the University of Paraná, Rua General Carneiro, 181. CEP 80.000–000, Curitiba, Paraná, Brasil; 3 Department of Clinical Medicine, Petrópolis Faculty of Medicine, Av. Barão do Rio Branco 1003 – Centro, CEP 25.685.000, Petrópolis, Rio de janeiro, Brazil

## Abstract

The authors present the high-resolution computed tomography findings of a patient with idiopathic pneumonia syndrome after bone marrow transplantation. The main finding consisted of extensive ground-glass opacities superimposed to mild interlobular septal thickening, resulting in the appearance termed "crazy-paving". Following the clinical, laboratorial and imaging criteria, the diagnosis of idiopathic pulmonary syndrome was defined and corticosteroids were introduced. The clinical symptoms improved in the following days, and the patient was discharged from the hospital.

## Background

Bone marrow transplantation (BMT) is an important treatment option for patients with haematological malignancies and severe disorders of the haematopoietic and immune systems. Infectious and non-infections pulmonary complications are common after BMT [1]. Idiopathic pneumonia syndrome (IPS) is included as one of the non-infectious complications that may occur after BMT [2]. This condition usually manifests between 42–49 days after BMT, and the clinical presentation includes dyspnea, dry cough and hypoxemia [3]. The chest X-ray frequently shows non-lobar infiltrates and the high-resolution CT presents multifocal reticular opacities and nodules of several sizes [3]. To our knowledge, considering the pulmonary complications after BMT, the "crazy-paving" pattern was not previously described in patients with idiopathic pneumonia syndrome.

The aim of this study is to report a case of IPS after-BMT presenting with "crazy-paving" pattern on the high-resolution computed tomography (CT) scan.

## Case presentation

A 51-year-old male patient presented with thoracic pain, dry cough and dyspnea in the 16th day after BMT for mielodisplastic syndrome. The lung auscultation demonstrated diffuse crackles. There were no clinical signs of acute GVHD. Various drugs were administered for possible pneumonia caused by viruses, bacteria, fungi, and Pneumocystis carinii, but these were ineffective. The arterial blood oxygen saturation was decreased. Cultures of sputum, blood, bronchoalveolar lavage, and urine for mycobacteria, fungi, and bacteria were negative. Serum titers against Mycoplasma pneumoniae, Chlamydia psittaci, and various viruses (cytomegalovirus, herpes simplex, varicella-zoster virus, adenovirus, influenza A, influenza B, and respiratory syncytial virus), were performed. All the serology and polymerase chain reaction tests were negative.

**Figure 1:**
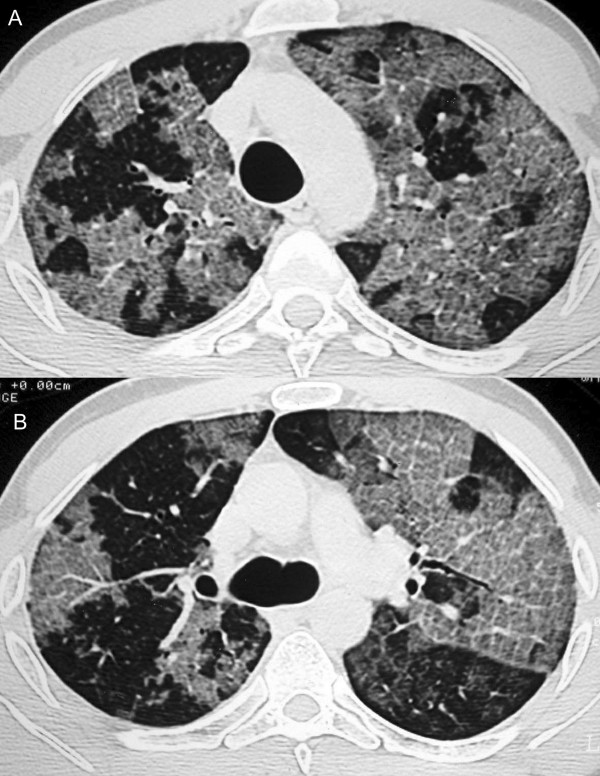
**A and B: **High resolution CT of the lungs demonstrates diffuse ground-glass attenuation with superimposed interlobular septal thickening ("crazy-paving" pattern), mainly in the superior lobes.

The chest X-ray findings were unremarkable. The high-resolution CT scan demonstrated a bilateral lesion, characterized by ground-glass opacities superimposed to interlobular septal thickening ("crazy-paving" pattern), predominating in the superior lobes (figure 1). Isolated areas of ground-glass attenuation were also seen in both lungs.

Following the clinical, laboratorial and imaging criteria established in the medical literature, the diagnosis of idiopathic pulmonary syndrome was defined and corticosteroids were introduced.

The respiratory symptoms improved in the following days. One week later, the high-resolution CT showed that the areas of "crazy-paving" pattern partially regressed. The patient discharged from the hospital taking only corticosteroids.

## Discussion

The IPS after BMT is defined as a diffuse lung injury, in which infectious or non-infectious aetiology are not identified [4]. The incidence of IPS after allogeneic BMT is approximately 12%. The median time of onset is 42–49 days after the procedure, but there is an early peak in the first 14 days, followed by another up to 80 days [5]. Multiple factors may contribute to this condition, including immunological defects secondary to underling disease and its treatment, conditioning regimen, and development of graft-versus-host disease (GVHD) [6]. The clinical presentation includes fever, dry cough, dyspnea and hypoxemia [5]. Clark et al [4] proposed the diagnosis criteria for IPS after BMT: symptoms and signs of pneumonia, evidence of abnormal pulmonary physiology (increased alveolar to arterial oxygen gradient or restrictive pattern on the pulmonary function tests), evidence of widespread alveolar injury suggested by chest X-ray or CT, and absence of active lower respiratory tract infection. The treatment is unspecific, and corticoids have not been improved mortality of this entity [7-9]. The prognosis is poor, with a case fatality of 74%. However, if the patient required mechanical ventilation, the mortality rate may exceed 95% [10]. In the present case, the patient presented with IPS 16 days after BMT and the diagnosis followed the Clark's criteria.

The radiological pattern of IPS consists of alveolar and interstitial infiltrates, with diffuse and bilateral distribution [7,11]. In a study with 31 cases of interstitial pneumonia, including 20 cases of IPS, the main radiographic and the high-resolution CT findings were diffuse multifocal alveolar opacities, reticular opacities, interlobular septal thickening, and nodules of several sizes [11].

The "crazy-paving" pattern at high-resolution CT of the lungs is characterized by scattered or diffuse ground-glass attenuation areas with superimposed interlobular septal thickening [3,12]. This finding had been considered suggestive of alveolar proteinosis, but it has subsequently been reported in a variety of infectious, neoplastic, idiopathic, inhalational, and sanguineous disorders of the lung [5,12]. In patients who underwent BMT and presented with pulmonary complications, the "crazy-paving" pattern was previous described in a case of pulmonary toxoplasmosis [13]. The initial high-resolution CT scan of our patient showed a bilateral and diffuse lesion, characterized by ground glass opacities associated with interlobular septal thickening ("crazy-paving" pattern).

## Conclusion

In conclusion, the IPS should be included in the differential diagnosis of pulmonary complications after BTM presenting "crazy-paving" pattern at high-resolution CT.

## Competing interests

The authors declare that they have no competing interests.

## Authors' contributions

TDG conceived the study. TDG, EM and MBG research the literature review and prepared the manuscript. DLE and GZ edit and coordinated the manuscript. All authors read and approved the final manuscript.

## Consent

Written informed consent was obtained from the patient for publication of this case report and accompanying images. A copy of the written consent is available for review by the Editor-in-Chief of this journal.
